# Factors contributing to discrepant estimated glomerular filtration values measured by creatinine and cystatin C in patients with rheumatoid arthritis

**DOI:** 10.1038/s41598-021-89303-3

**Published:** 2021-05-10

**Authors:** Akikatsu Nakashima, Shigeto Horita, Takahiro Matsunaga, Ryo Inoue, Takeshi Zoshima, Ichiro Mizushima, Satoshi Hara, Kiyoaki Ito, Hiroshi Fujii, Hideki Nomura, Mitsuhiro Kawano

**Affiliations:** 1 Division of Nephrology and Rheumatology, Ishikawa Prefectural Central Hospital, Kanazawa, Japan; 2 Division of Internal Medicine and Rheumatology, Ishikawa-Ken Saiseikai Kanazawa Hospital, Kanazawa, Japan; 3 Department of Rheumatology, Kanazawa University Hospital, 13-1, Takaramachi, Kanazawa, Ishikawa 920-8641 Japan; 4 Department of General Medicine, Kanazawa University Hospital, Kanazawa, Japan

**Keywords:** Biomarkers, Rheumatology

## Abstract

This study aimed to clarify the factors underlying the discrepancy that has been noted between estimated glomerular filtration ratio (eGFR) measured using serum creatinine (Cr) and eGFR using serum cystatin C (CysC) in patients with rheumatoid arthritis (RA) and to identify those patients whose renal function should be evaluated using CysC. We retrospectively evaluated clinical features, disease activity, Steinbrocker radiological staging, and co-morbidities (diabetes mellitus, hypertension, dyslipidemia) in 238 RA patients. eGFR using serum creatinine (eGFR-Cr) and eGFR using serum cystatin C (eGFR-CysC) were calculated using the new Japanese coefficient-modified Modification of Diet in Renal Disease study equation. To clarify the cause(s) of differences of 20% or more between the two eGFRs, we divided our RA patients into Group A (eGFR-Cr/eGFR-CysC ≥ 1.2) and Group B (eGFR-Cr/eGFR-CysC < 1.2), and searched for factors independently related to Group A. Forty-five patients (18.9%) were assigned to Group A, and 193 (81.1%) to Group B. BMI (Odds Ratio [OR] 0.820, 95% confidence interval [CI] 0.675–0.996), Hb (OR 0.633, 95% CI 0.433–0.926), CK (OR 0.773 per 10 units, 95% CI 0.644–0.933), NSAID use (OR 0.099, 95% CI 0.020–0.494), diabetes mellitus (OR 6.024, 95% CI 1.508–24.390) and stage 4 Steinbrocker radiological stage (OR 10.309, 95% CI 2.994–35.714) were identified as independent relevant factors for Group A by a multifactorial analysis. Renal function in RA patients with low BMI, diabetes, anemia and low CK may be overestimated using eGFR-Cr alone, and such patients need to be evaluated using eGFR-CysC.

## Introduction

The therapeutic paradigm of rheumatoid arthritis (RA) has changed dramatically with the advent of biological disease modifying anti-rheumatic drugs (bDMARDs). In addition to conventional synthetic DMARDs (csDMARDs) such as methotrexate (MTX), bDMARDs that selectively target each cytokine have emerged. Recently, drugs that target small molecules such as Janus kinase (JAK) in cells have been used as targeted synthetic DMARDs (tsDMARDs) as effectively as bDMARDs^1^. One of these drugs, MTX, is known to cause adverse effects such as cytopenia when administered to patients with renal dysfunction, and is contraindicated in patients with estimated glomerular filtration rate (eGFR) less than 30 mL/min/1.73 m^2^^2^. Baricitinib, a JAK inhibitor, is also contraindicated in these patients due to its renal excretion^3^. Thus, accurate evaluation of renal function is mandatory to ensure the safest and most effective treatment for RA with DMARDs.

On the other hand, 8–25% of RA patients have been reported to have reduced renal function (eGFR < 60 mL/min/1.73 m^2^)^4–7^. Factors such as inflammation, use of various therapeutic drugs, primary glomerular diseases, and amyloidosis are considered to contribute to this renal dysfunction^8,9^.

In regard to the evaluation of renal function, eGFR using the serum creatinine level (eGFR-Cr) is used in daily clinical practice, but has been reported to be overestimated in RA patients because of their low muscle mass^10^. Serum cystatin C (CysC) is a low molecular weight protein (13 kilodalton), which is generally produced at a constant rate from nucleated cells, and reabsorbed and catabolized in the proximal renal tubule after being filtered through the glomeruli. Thus, CysC has drawn attention as a marker of renal function independent of muscle mass^11^, and an eGFR calculated from the serum CysC level (eGFR-Cys) has also been developed as an alternative parameter^12^.

Although it has long been presumed that a discrepancy exists between eGFR-Cr and eGFR-CysC in RA patients, neither the frequency of any such discrepancy nor the factor(s) causing it have been investigated. This state of affairs prompted us to undertake the present study to search for factors that might explain the discrepancy between eGFR-Cr and eGFR-CysC in RA patients and determine the clinical features of patients whose renal function should be evaluated using CysC.

## Methods

We conducted a cross-sectional observational study. The study was conducted in strict accordance with the principles of the Declaration of Helsinki and the Japanese ethical guidelines for clinical research and approved by the Ethics Committee of Ishikawa Prefectural Central Hospital (approval no. 1314) and the Medical Ethics Committee of Kanazawa University (approval no. 1516-2). Informed consent was obtained from all patients.

### Patients

We enrolled 238 RA outpatients treated from December 2018 to January 2019 from Ishikawa Prefectural Central Hospital and Kanazawa University Hospital. All patients fulfilled the 1987 American College of Rheumatology (ACR) classification criteria for RA^13^ and/or the ACR/European League Against Rheumatism 2010 classification criteria for RA^14^.

### Data collection and grouping of patients

We measured complete blood cell counts, erythrocyte sedimentation rate (ESR), and serum levels of Cr, blood urea nitrogen (BUN), uric acid (UA), total protein, albumin (Alb), aspartate transaminase (AST), alanine aminotransferase (ALT), lactate dehydrogenase (LDH), creatine kinase (CK), C-reactive protein (CRP), immunoglobulin (Ig) G, IgA, IgM, complement 3 (C3), C4, total hemolytic complement (CH50), rheumatoid factor (RF), anti-cyclic citrullinated peptide antibodies (aCCP), and CysC. Swollen joints, tender joints, patient visual analogue scale (VAS), and physician VAS were evaluated based on the physical findings. In addition, clinical disease activity index (CDAI), simplified disease activity index (SDAI), disease activity score (DAS) 28 using ESR (DAS28-ESR), and DAS28 using CRP (DAS28-CRP) were calculated. Urine protein and occult blood were evaluated with dipsticks. Presence or absence of smoking history and comorbidities such as diabetes mellitus, hypertension and hyperlipidemia were derived from the medical records. Medication for RA, including non-steroid anti-inflammatory drugs (NSAIDs), prednisolone, csDMARD (MTX, salazosulfapyridine, bucillamine, and tacrolimus), bDMARDs (infliximab, etanercept, adalimumab, tocilizumab, abatacept, golimumab and certolizumab pegol), and tsDMARDs (tofacitinib and baricitinib) were evaluated. The radiological stage and functional classification of Steinbrocker were evaluated^15^. Patients were divided into stage 4 and other stages, and class 4 and other classes.

Body surface area indexed (BSA-indexed) eGFR-Cr and eGFR-Cys were calculated based on the following formula: eGFR-Cr (mL/min/1.73 m^2^) = 194 × (serum Cr)^−1.094^ × (age)^−0.287^ (× 0.739, when female)^16^, and eGFR-CysC (mL/min/1.73 m^2^) = 104 × (serum CysC)^−1.019^ × 0.996^(age)^ (× 0.929, when female) − 8^12^, respectively. BSA was calculated using Du Bois's formula: BSA (m^2^) = 0.007184 × [weight (kg)]^0.425^ × [height (cm)]^0.725^^17^, and absolute eGFR-Cr and eGFR-CysC were calculated based on these parameters. Based on the eGFR-Cr and eGFR-CysC, we divided the patients into Group A (eGFR-Cr/eGFR-CysC ≥ 1.2), whose eGFR-Cr was more than 20% higher than eGFR-CysC, and Group B (eGFR-Cr/eGFR-CysC < 1.2), and compared the above-noted parameters between them.

### Statistics

The results were expressed as mean ± standard deviation. In the univariate analysis, the significance of differences between the mean values of the two groups was determined using the Mann–Whitney test for continuous data. The significance of differences in frequencies was tested using χ^2^ or Fisher's exact test for categorical data as appropriate. A histogram of eGFR-Cr/eGFR-CysC with a logarithmic scale on the X axis was made. Pearson's correlation coefficient was used for testing the correlation between the two variates. For multivariate analysis to search for independent factors related to Group A, binomial logistic regression analysis was performed by the forward stepwise method. In addition, the Hosmer–Lemeshow test was used to confirm the fitness of the model^18^. P values of the two-tailed test less than 0.05 were considered to be significant. All statistics were performed by using SPSS 26.0 software.

## Results

### Demographics of RA patients

A total of 238 RA patients (190 women, mean age: 65.3 ± 14.0 years, disease duration: 12.0 ± 11.0 years) were included (Table 1). Smoking habit was present in 56 patients (23.5%), hypertension in 83 (34.9%), hyperlipidemia in 44 (18.5%), and diabetes mellitus in 39 (16.4%). Proteinuria was observed in 17 (7.1%) and hematuria in 48 (20.2%). Almost all patients were outpatients in remission or a low disease activity state based on the various disease activity parameters.

**Table 1:** Characteristics of the subjects.

	Mean ± SD	Range
Age (years)	65.3 ± 14.0	27–94
Female/male	190/48	
Disease duration (years)	12.0 ± 11.0	0–63
Height (cm)	156.8 ± 8.6	136–180
Body weight (kg)	54.3 ± 11.3	31.1–98.0
Body mass index	22.0 ± 3.6	13.9–37.0
Smoking, n (%)	56 (23.5%)	
Hypertension, n (%)	83 (34.9%)	
Hyperlipidemia, n (%)	44 (18.5%)	
Diabetes mellitus, n (%)	39 (16.4%)	
Proteinuria, n (%)	17 (7.1%)	
Hematuria, n (%)	48(20.2%)	
Serum creatinine (mg/dL)	0.72 ± 0.28	0.33–2.97
eGFR-Cr (mL/min/1.73 m^2^)	72.5 ± 20.2	12.0–142.0
Absolute eGFR-Cr (mL/min)	64.1 ± 19.7	10.0–130.5
Serum Cystatin C (mg/dL)	1.03 ± 0.43	0.49–5.24
eGFR-CysC (mL/min/1.73 m^2^)	73.2 ± 24.9	6.3–167.8
Absolute eGFR-CysC (mL/min/1.73 m^2^)	65.3 ± 24.4	4.8–153.6
DAS28-CRP	2.0 ± 0.9	1.0–6.4
DAS28-ESR	2.7 ± 1.1	0.5–7.5
CDAI	4.7 ± 6.3	0–46
SDAI	5.0 ± 6.5	0.0–47.9
RF (U/mL)	137.1 ± 356.0	0–3720
ACPA positive, n (%)	135/188 (71.8%)	27–94
Methotrexate, n (%)	120 (50.4%)	
Dose (mg/week)	7.5 ± 2.7	2–14
Prednisolone, n (%)	109 (45.8%)	
Dose (mg/day)	3.8 ± 2.3	1–10
NSAIDS, n (%)	65 (27.3%)	
Tacrolimus, n (%)	65 (27.3%)	
Salazosulfapyridine, n (%)	25 (10.5%)	
Biologic therapy, n (%)	69 (29.0%)	
JAK inhibitors, n (%)	3 (1.3%)	

*eGFR-Cr* body surface area (BSA)-indexed estimated glomerular filtration rate using creatinine, *Absolute eGFR-Cr* absolute estimated glomerular filtration rate using creatinine, *eGFR-CysC* body surface area (BSA)-indexed estimated glomerular rate using cystatin C, *Absolute eGFR-CysC* absolute estimated glomerular filtration rate using cystatin C, *DAS28-CRP* disease activity score, using the 28-joint count and C reactive protein, *DAS28-ESR* disease activity score, using the 28-joint count and erythrocyte sedimentation rate, *CDAI* clinical disease activity index, *SDAI* simplified disease activity index, *RF* rheumatoid factor, *ACPA* anti-cyclic citrullinated peptide antibody, *NSAIDS* non-steroidal anti-inflammatory drugs, *JAK Inhibitors* Janus kinase inhibitor.

For medication, MTX was administered to 120 patients (50.4%) at an average dose of 7.5 ± 2.7 mg/week, prednisolone to 109 (45.8%) at 3.8 ± 2.3 mg/day, NSAIDs to 65 (27.3%), and tacrolimus to 65 (27.3%). bDMARDS were administered to 69 patients (29.0%), and tsDMARDs to 3 (1.3%).

**Figure 1:**
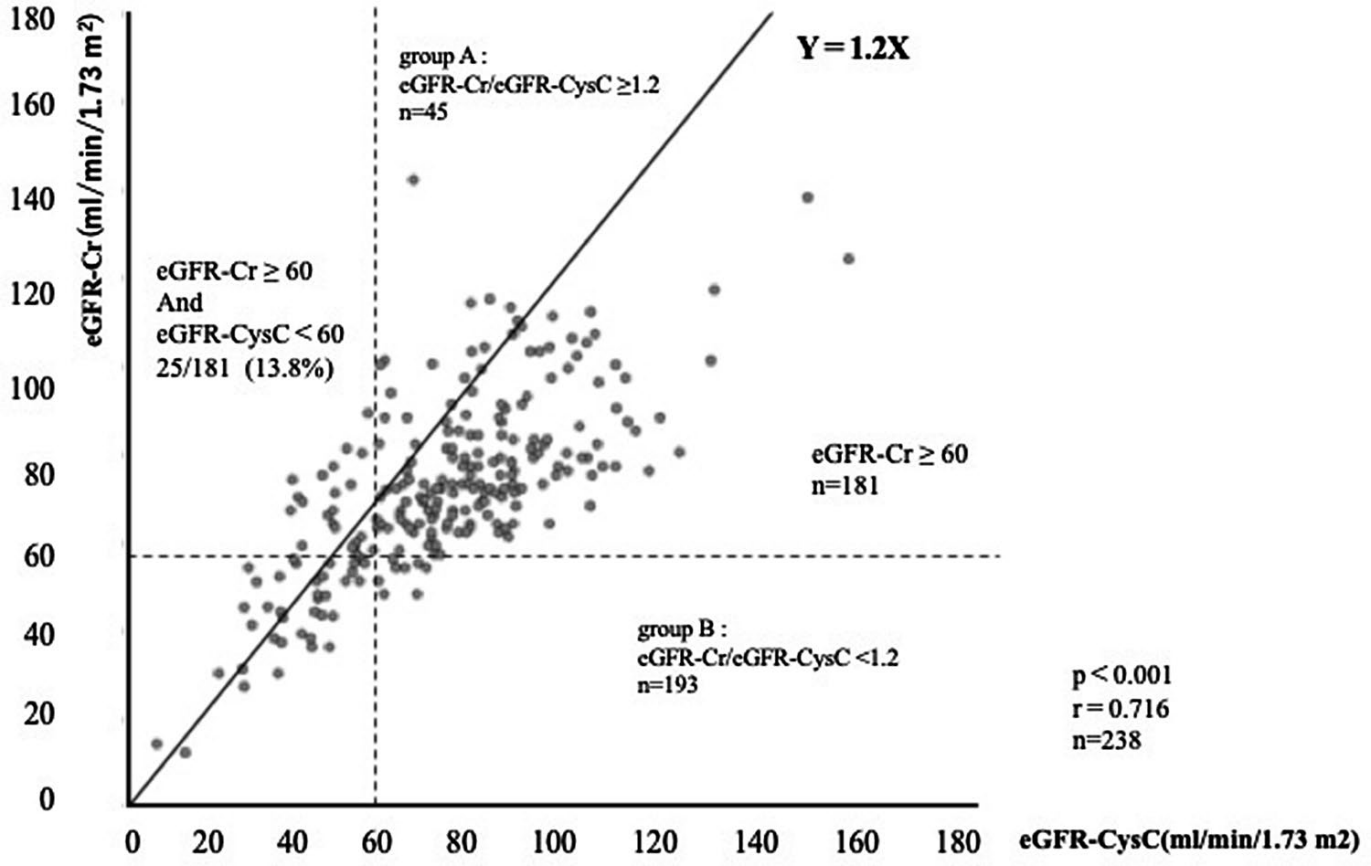
Relationship between eGFR-Cr and eGFR-CysC. eGFR-Cr; estimated glomerular filtration rate using creatinine. eGFR-CysC; estimated glomerular filtration rate using cystatin C. The correlation between eGFR-Cr and eGFR-CysC was significant (r = 0.715, p < 0.0001). However, 25 (13.8%) of 181 patients with eGFR-Cr ≥ 60 mL/min/1.73 m^2^ showed eGFR-Cys < 60 mL/min/1.73 m^2^.

The average values of eGFR-Cr and eGFR-CysC were 72.5 ± 20.2 (12.0–142.0) and 72.3 ± 24.9 (6.3–167.8) mL/min/1.73 m^2^, respectively, with this difference not significant. The correlation between them was significant (r = 0.715, p < 0.0001). However, 25 (13.8%) of 181 patients with eGFR-Cr ≥ 60 mL/min/1.73 m^2^ showed eGFR-CysC < 60 mL/min/1.73 m^2^ (Fig. 1).

The number of patients with absolute-eGFR-Cr < 60 and ≥ 60 mL/min were 99 and 139, respectively. Eighteen (12.9%) of 139 patients with absolute eGFR-Cr ≥ 60 mL/min showed absolute eGFR-CysC < 60 mL/min.

**Figure 2:**
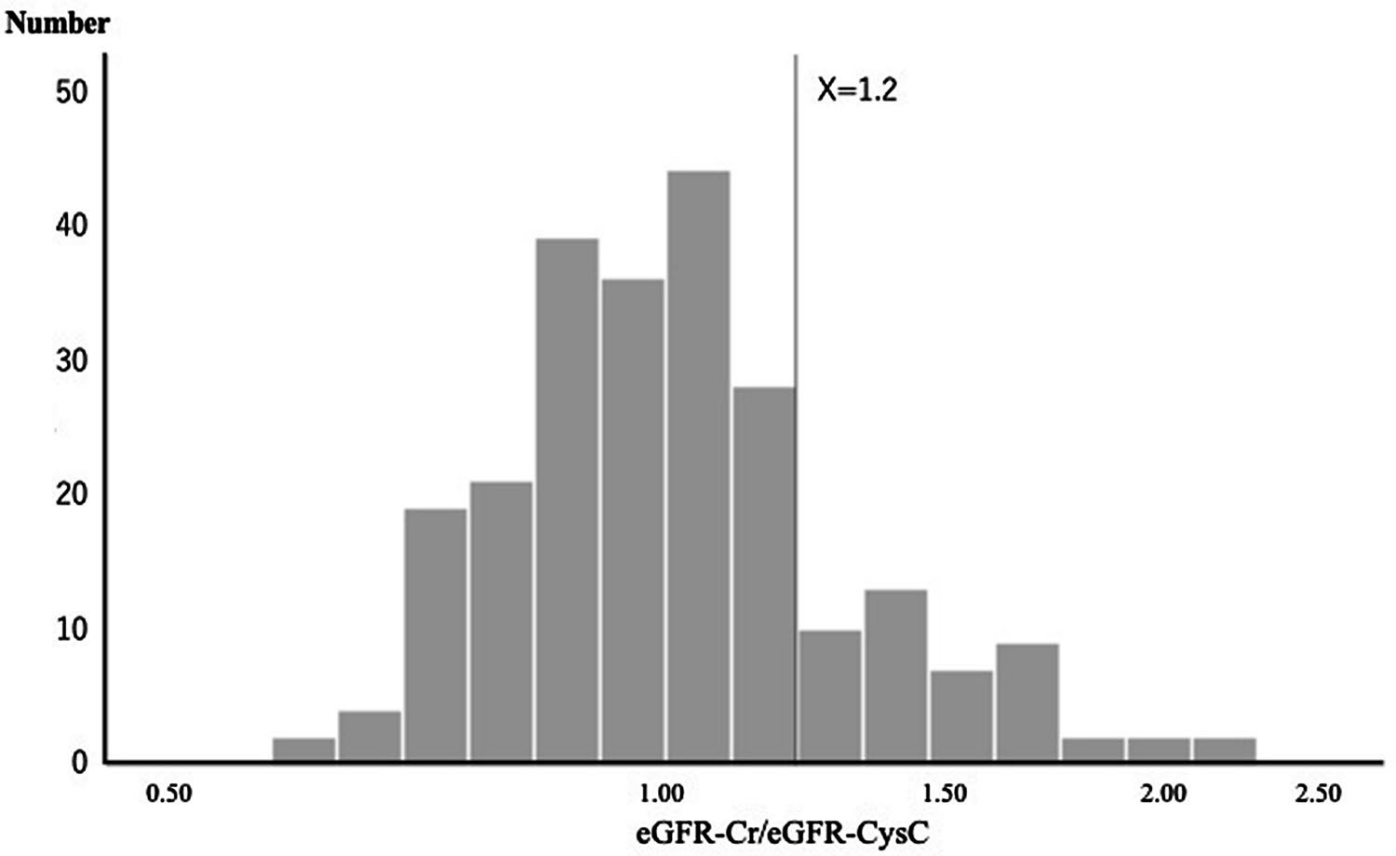
Distribution of eGFR-Cr/eGFR-CysC using the histogram with a logarithmic scale on the X axis. eGFR-Cr; estimated glomerular filtration rate using creatinine. eGFR-CysC; estimated glomerular filtration rate using cystatin C. There was an inflection point at 1.2. Thus, we divided the patients into two groups by this point (greater than 20% discrepancy).

Subsequently, we examined the distribution of the ratio of eGFR-Cr and eGFR-CysC using a histogram with a logarithmic scale on the X axis (Fig. 2), and found an inflection point at 1.2. With reference to this histogram of eGFR-Cr/eGFR-CysC, the nephrologists and rheumatologists in our team reached the consensus that values of eGFR-Cr/eGFR-CysC of ≥ 1.2 might be of clinical concern in the management of chronic kidney disease. Thus, we divided the patients into two groups by this point (greater than 20% discrepancy). Forty-five patients (18.9%) with eGFR-Cr/eGFR-CysC ≥ 1.2 were categorized as Group A (n = 45), and 193 (81.1%) with eGFR-Cr/eGFR-CysC < 1.2 as Group B.

### Univariate statistical analysis

Compared with the patients in Group B, those in Group A were older (73.8 ± 12.5 vs 63.2 ± 13.6 years), and had a longer disease duration (17.7 ± 14.0 vs 10.4 ± 9.5 years), lower body mass index (BMI) (20.0 ± 2.9 vs 22.4 ± 3.6 kg/m^2^), higher frequencies of hypertension and diabetes mellitus (55.6% vs 30.1% and 35.6% vs 11.0%, respectively), higher disease activity (DAS28-ESR 3.1 ± 1.3 vs 2.6 ± 1.0), lower hemoglobin (Hb) (11.8 ± 1.8 vs 12.8 ± 1.4 g/dL), higher CRP (0.56 ± 0.79 vs 0.27 ± 0.50 mg/dL), higher ESR (43 ± 31 vs 24 ± 21 mm/h), lower CK (63.9 ± 36.0 vs 92 ± 79 IU/L), and higher frequencies of Steinbrocker stage 4 and class 4 (47.7% vs. 16.1% and 22.7% vs 1.1%, respectively) (Table 2). With regard to medication, Group A had a lower frequency of MTX use (35.6% vs 53.9%) with lower dose (6.3 ± 2.5 vs 7.2 ± 2.7 mg/week), a higher frequency of prednisolone use (66.7% vs 40.9%) with higher dose (4.8 ± 2.6 vs 3.3 ± 2.1 mg/day), and a lower frequency of NSAIDs (13.3% vs 30.6%). No differences were found in the use of either bDMARDs or tsDMARDs.

**Table 2:** Differences in parameters between Group A and Group B.

Parameter (units)	Group A (n = 45)	Group B (n = 193)	p value
Age (years)	73.8 ± 12.5	63.3 ± 13.6	< 0.001
Female/male	35/10	155/38	0.684
Disease duration (years)	17.3 ± 14.1	10.6 ± 9.7	0.001
Body mass index (kg/m^2^)	20.0 ± 2.9	22.4 ± 3.6	< 0.001
Hypertension, n (%)	25/45 (55.6%)	58/193 (30.1%)	0.002
Hyperlipidemia, n (%)	10/45 (22.2%)	34/193 (17.6%)	0.523
Diabetes mellitus, n (%)	16 (35.6%)	23/193 (11.9%)	< 0.001
Proteinuria, n (%)	3/45 (6.7%)	14/193 (7.3%)	0.831
Hematuria, n (%)	6 (13.3%)	42/193 (21.8%)	0.366
DAS28-CRP	2.2 ± 1.1	1.9 ± 0.9	0.189
DAS28-ESR	3.1 ± 1.3	2.6 ± 1.0	0.013
RF (U/mL)	156.2 ± 234.4	132.6 ± 379.4	0.178
ACPA positive, n (%)	22/33 (66.7%)	113/155 (72.9%)	0.554
WBC (/mm^3^)	6422 ± 2848	6086 ± 2112	0.582
Hb (g/dL)	11.8 ± 1.8	12.8 ± 1.4	< 0.001
Plts. (× 10^4^/mm^3^)	23.2 ± 7.2	23.5 ± 7.3	0.736
CRP (mg/dL)	0.56 ± 0.79	0.27 ± 0.50	0.010
ESR (mm/1 h)	43 ± 31	24 ± 21	< 0.001
Albumin (g/dL)	3.7 ± 0.5	4.1 ± 0.4	< 0.001
BUN (mg/dL)	18.4 ± 6.1	15.7 ± 4.9	0.004
Cr (mg/dL)	0.71 ± 0.39	0.73 ± 0.25	0.037
CysC (mg/L)	1.37 ± 0.70	0.95 ± 0.30	< 0.001
eGFR-Cr (mL/min/1.73 m^2^)	76.7 ± 25.6	71.5 ± 18.6	0.158
eGFR-CysC (mL/min/1.73 m^2^)	52.5 ± 19.9	78.1 ± 23.4	< 0.001
CK (IU/L)	64 ± 36	92 ± 79	0.001
Steinbrocker Stage 4 (n/%)	21/44 (47.7%)	31/192 (16.1%)	< 0.001
Steinbrocker Class 4 (n/%)	10/44 (22.7%)	2/190 (1.1%)	< 0.001
Methotrexate (n/%)	16/45 (35.6%)	104/193 (53.9%)	0.032
Prednisolone (n/%)	30/45 (66.7%)	79/193 (40.9%)	0.003
NSAIDs (n/%)	6/45 (13.3%)	59/193 (30.6%)	0.025

*Group A* eGFR-Cr/eGFR-CysC ≥ 1.2, *Group B* eGFR-Cr/eGFR-CysC < 1.2), *DAS-CRP* disease activity score, using the 28-joint count and C reactive protein, *DAS-ESR* disease activity score, using the 28-joint count and erythrocyte sedimentation rate, *CDAI* clinical disease activity index, *SDAI* simple disease activity index, *RF* rheumatoid factor, *ACPA* anti-cyclic citrullinated peptide antibody, *eGFR-Cr* estimated glomerular rate using creatinine, *eGFR-CysC* estimated glomerular rate using cystatin C.

### Multivariate statistical analysis: logistic regression

Multivariate binary logistic regression analysis with a forward stepwise method was performed to detect factors related to Group A (Table 3). Hosmer–Lemeshow test confirmed the fitness of the model (p = 0.683). BMI [per kg/m^2^, Odds ratio (OR) 0.820, 95% confidence interval (95% CI) 0.675–0.996], Hb (per g/dL, OR 0.633, 95% CI 0.433–0.926), CK (per 10 units, OR 0.773, 95% CI 0.644–0.933), use of NSAIDs (OR 0.099, 95% CI 0.020–0.494), diabetes mellitus (OR 6.024, 95% CI 1.508–24.390), Steinbrocker stage 4 (vs other stages, OR 10.309, 95% CI 2.994–35.714) were found to be significantly related to Group A.

**Table 3:** Logistic analysis by forward stepwise method for Group A and Group B.

	P value	Odds ratio (95% confidence interval)
Age (years)	0.836	1.005 (0.961–1.052)
Gender	0.273	2.304 (0.518–10.204)
BMI (kg/m^2^)	0.045	0.820 (0.675–0.996)
Hb (g/dL)	0.018	0.633 (0.433–0.926)
CK (10 units)	0.006	0.773 (0.644–0.933)
NSAIDS	0.005	0.099 (0.020–0.494)
Diabetes mellitus	0.011	6.024 (1.508–24.390)
Steinbrocker Stage 4	0.001	10.309 (2.994–35.714)

Hosmer Lemeshow Test p = 0.683.

*BMI* body mass index, *Hb* hemoglobin, *CK* creatine phosphokinase, *NSAIDS* non-steroidal anti-inflammatory drugs.

A multinomial logistic regression with a dependent variable composed of three eGFR-Cr/eGFR-CysC categories, namely groups A (1.2 ≤ eGFR-Cr/eGFR-CysC), B1 (0.8 ≤ eGFR-Cr/eGFR-CysC < 1.2) and B2 (eGFR-Cr/eGFR-CysC < 0.8), revealed that younger age and Steibbrocker radiological stages 1–3 were the only predictive factors for group B2 when compared to group B1 while the comparison between groups A and B1 showed similar results between group A and B, (Supplementary Table 1 and 2). Thus, the significance of group B2 as a separate group was considered to be insignificant clinically.

Estimated odds ratios of 6 factors were integrated for each patient, and the patient group was divided into 10 quantiles according to the value. Of the 23 patients with the highest predicted odds ratio, thirteen had eGFR-Cr/eGFR-Cysc > 1.2, and of 23 patients with the lowest predicted, one patient did, resulting in an actual odds ratio of 28.6. In addition, the mean eGFR-Cr/eGFR-CysC for the highest-ranked patients was 1.38 (up to 2.22), and the mean for the lowest-ranked patients was 0.96.

## Discussion

We showed that eGFR-Cr was more than 20% higher than eGFR-CysC in 18.9% of our cohort of RA patients. Furthermore, an overestimation of renal function when using eGFR-Cr was significantly related to lower BMI, lower Hb, lower CK, no NSAIDs use, diabetes mellitus, and Steinbrocker radiological stage 4.

The most accurate measure of GFR is considered to be inulin clearance (Cin). However, it is impractical to measure Cin in all patients in daily clinical practice, and estimated GFR (eGFR) is resorted to instead. Nozawa et al.^19^ compared Cin with eGFR using the serum creatinine (eGFRcreat), eGFR using the serum cystatin C concentration (eGFRcys), the mean of eGFRcreat, eGFRcys (eGFRavg) and endogenous 24-h creatinine clearance × 0.715 (Ccr × 0.715) in 30 RA patients with a daily prednisolone dose less than 10 mg and demonstrated that eGFRcys was correlated best with Cin, and that the relationship was close to y = x. It has been reported that serum cystatin C concentration is affected by factors such as steroid dose^19^, disease activity^20,21^, and thyroid function^22^. However, in this study, most of the patients were receiving less than prednisolone 10 mg daily, and patients with moderate to high disease activity according to DAS28CRP were 7 (15.5%) in group A and 20 (10.3%) in group B, representing relatively small numbers. And patients with thyroid disease that affects CysC were not included into our study. There were almost no known factors that affect eGFR-CysC in our study, and thus our research was conducted on the assumption that eGFR-CysC could be used as an accurate surrogate marker of Cin.

An overestimation of renal function may occur when using eGFR-Cr for evaluation of renal function in patients with RA. In our study, a significant positive correlation was observed between eGFR-Cr and eGFR-CysC. However, 13.8% of patients with eGFR-Cr ≥ 60 mL/min/1.73 m^2^ showed eGFR-CysC < 60 mL/min/1.73 m^2^. Therefore, these patients may be at risk of having chronic kidney disease (CKD) overlooked and being treated as patients with normal renal function. This may lead to unexpected adverse effects when renally excreted drugs are used.

Lin et al.^23^ reported that myopenia (sarcopenia) is very common in RA patients and associated with functional limitations and joint disorders. Torii et al.^24^ found myopenia to be independently associated with age, long duration of illness, joint destruction, and malnutrition in RA. In our study, lower BMI and Steinbrocker stage 4 were identified as independent factors relevant to Group A, suggesting that Group A included more patients with myopenia. Umegaki et al.^25^ and Scott et al.^26^ reported that diabetic patients frequently had sarcopenia. In our study, diabetes mellitus was related to Group A, suggesting an association with sarcopenia. In recent years, the treatment of RA has progressed dramatically with the advent of bDMARDs and tsDMARDs, and the number of RA patients with myopenia has decreased. Nevertheless, our study documented a possible overestimation of renal function by more than 20% with eGFR-Cr in about 19% of RA patients, which was likely attributable to the presence of myopenia/sarcopenia. Flahault et al.^27^ reported that low serum CK levels in CKD patients reflected loss of muscle mass and malnutrition and were associated with high mortality. In our study, low CK was found to be an independent factor relevant to Group A. This low CK was also considered to be associated with sarcopenia. According to a report on the relationship between low CK and sarcopenia^27^ and another on the relationship between BMI and muscle mass^28^, considering the difficulty of measuring actual muscle mass using Body Composition^23^ for many patients, we evaluated BMI and CK as surrogate markers for muscle mass.

Horio et al.^29^ reported that hypoalbuminemia was significantly associated with elevated eGFR/Cin levels, due to increased Cr secretion from the renal tubules and decreased creatinine production because of low muscle mass. In our study, univariate analysis showed that group A had significantly lower albumin levels than group B (3.7 ± 0.5 vs 4.1 ± 0.4, p < 0.001), but multivariate analysis did not demonstrate lower albumin levels to be an independent relevant factor for group A. The results of the univariate analysis revealed that the overestimation of eGFR in group A was attributable to the effects of hypoalbuminemia. Low CK level was an independent relevant factor for group A on multivariate analysis. Low CK level reflects low muscle mass, and so considering the association between low serum albumin levels and low muscle mass, for the multivariate analysis the more influential CK was selected instead of the less influential albumin.

Some studies have reported that chronic inflammation causes renal dysfunction in RA patients. Kochi et al.^30^ showed that persistently elevated CRP was related to CKD. Sumida et al.^31^ reported that the use of bDMARDs reduced the risk of developing CKD, and suggested that controlling chronic inflammation might contribute to prevention of CKD. On the other hand, Couderc et al.^32^ studied the risk factors of eGFR < 60 mL/min/1.73 m^2^ in 931 RA patients retrospectively, and reported that disease duration and disease activity (DAS28-ESR) were not related factors. In our study, univariate analysis showed Group A to have higher DAS28-ESR, CRP, and ESR than Group B, but multivariate analysis did not find them significant factors. Alternatively, our multivariate analysis indicated that anemia was associated with Group A. Masson et al.^33^ reported hepcidin and interleukin-6 to be important relevant factors for anemia in RA. Thus, in our analysis, though disease activity and inflammatory markers were not found to be independent relevant factors, anemia, which could be caused by inflammation, was related to the discrepancy between eGFR-Cr and eGFR-CysC. It was suggested that disease activity and inflammatory markers may promote muscle mass reduction mediated by inflammation.

Nozawa et al.^19^ noted that eGFR-CysC appeared to be lower when ≥ 10 mg/day prednisolone was administered, while not being affected by < 10 mg. In our study, Group A used prednisolone ≤ 10 mg/day (3 patients administered 10 mg/day prednisolone), and thus it was considered to have a negligible effect on eGFR-CysC.

Less frequent administration of NSAIDs was found to be related to Group A. Even though serum Cr values were similar, as Group A had higher serum CysC and BUN levels, they were judged to have clinical impairment of renal function. Thus, MTX and NDAIDs were less used in Group A. It was unlikely that medication for RA affected directly the discrepancy between eGFR-Cr and eGFR-CysC.

This study showed that 23.9% of the patients had eGFR-Cr < 60 mL/min/1.73 m^2^, and 41.6% had absolute eGFR-Cr < 60 mL/min. Saisho et al.^34^ examined eGFR-Cr of 7135 RA patients in the Ninja study from Japan, and reported that the values of eGFR-Cr from 60 to 30, 30 to 15, and < 15 mL/min/1.73 m^2^ were 17.5%, 0.8%, and 0.2%, respectively. Furthermore, Mori et al.^35^ reported from Japan that eGFR-Cr < 60 mL/min/1.73 m^2^ and absolute eGFR-Cr < 60 mL/min in RA patients were 18.6% and 33.8%, respectively. The results of these previous reports were consistent with ours with respect to BSA-indexed eGFR and absolute eGFR.

Our study had the following limitations. First, the numbers of patients might not have been sufficient. Second, outpatients with relatively stable disease control do not represent the entire RA population in terms of disease activity. Third, it was a temporary cross-sectional study. Fourth, we did not measure inulin clearance in our study.

## Conclusion

Renal function of RA patients with low BMI, diabetes mellitus, Steinbrocker X-ray joint destruction stage 4, anemia or lower CK can be overestimated when judged from eGFR-Cr alone. These patients require special attention, especially using eGFR-CysC, for accurately evaluating renal function.

## Supplementary Information

Supplementary Tables.

## Data Availability

All of the data supporting the conclusions of this article are included within the article. The authors declare that data will be made available upon request.

## References

[1] Köhler BM, Günther J, Kaudewitz D, Lorenz HM (2019). Current therapeutic options in the treatment of rheumatoid arthritis. J. Clin. Med..

[2] Boey O, Van Hooland S, Woestenburg A, Van der Niepen P, Verbeelen D (2006). Methotrexate should not be used for patients with end-stage kidney disease. Acta Clin. Belg..

[3] Zhang X (2017). Dose/exposure-response modeling to support dosing recommendation for phase III development of baricitinib in patients with rheumatoid arthritis. CPT Pharmacomet. Syst. Pharmacol..

[4] Karie S (2008). Kidney disease in RA patients: Prevalence and implication on RA-related drugs management: the MATRIX study. Rheumatology (Oxford).

[5] Daoussis D (2010). Cardiovascular risk factors and not disease activity, severity or therapy associate with renal dysfunction in patients with rheumatoid arthritis. Ann. Rheum. Dis..

[6] Daoussis D (2011). Microalbuminuria in rheumatoid arthritis in the post penicillamine/gold era: association with hypertension, but not therapy or inflammation. Clin. Rheumatol..

[7] Hickson LJ, Crowson CS, Gabriel SE, McCarthy JT, Matteson EL (2014). Development of reduced kidney function in rheumatoid arthritis. Am. J. Kidney Dis..

[8] Nakano M (1998). Analysis of renal pathology and drug history in 158 Japanese patients with rheumatoid arthritis. Clin. Nephrol..

[9] Helin HJ, Korpela MM, Mustonen JT, Pasternack AI (1995). Renal biopsy findings and clinicopathologic correlations in rheumatoid arthritis. Arthritis Rheum..

[10] Levey AS, Inker LA, Coresh J (2014). GFR estimation: From physiology to public health. Am. J. Kidney Dis..

[11] Laterza OF, Price CP, Scott MG (2002). Cystatin C: An improved estimator of glomerular filtration rate. Clin. Chem..

[12] Horio M (2013). GFR estimation using standardized serum cystatin C in Japan. Am. J. Kidney Dis..

[13] Arnett FC (1988). The American Rheumatism Association 1987 revised criteria for the classification of rheumatoid arthritis. Arthritis Rheum..

[14] van der Linden MP, Knevel R, Huizinga TW, van der Helm-van Mil AH (2011). Classification of rheumatoid arthritis: Comparison of the 1987 American College of Rheumatology criteria and the 2010 American College of Rheumatology/European League Against Rheumatism criteria. Arthritis Rheum..

[15] Steinbrocker O, Traeger CH, Batterman RC (1949). Therapeutic criteria in rheumatoid arthritis. J. Am. Med. Assoc..

[16] Matsuo S (2009). Revised equations for estimated GFR from serum creatinine in Japan. Am. J. Kidney Dis..

[17] Du Bois D, Du Bois EF (1989). A formula to estimate the approximate surface area if height and weight be known .1916. Nutrition.

[18] Hosmer DW, Wang CY, Lin IC, Lemeshow S (1978). A computer program for stepwise logistic regression using maximum likelihood estimation. Comput. Programs Biomed..

[19] Nozawa Y (2018). Utility of estimated glomerular filtration rate using cystatin C and its interpretation in patients with rheumatoid arthritis under glucocorticoid therapy. Clin. Chim. Acta.

[20] Targońska-Stepniak B, Majdan M (2011). Cystatin C concentration is correlated with disease activity in rheumatoid arthritis patients. Scand. J. Rheumatol..

[21] Sato H (2010). Cystatin C is a sensitive marker for detecting a reduced glomerular filtration rate when assessing chronic kidney disease in patients with rheumatoid arthritis and secondary amyloidosis. Scand. J. Rheumatol..

[22] Manetti L (2005). Thyroid function differently affects serum cystatin C and creatinine concentrations. J. Endocrinol. Investig..

[23] Lin JZ (2019). Myopenia is associated with joint damage in rheumatoid arthritis: a cross-sectional study. J. Cachexia Sarcopenia Muscle.

[24] Torii M (2019). Prevalence and factors associated with sarcopenia in patients with rheumatoid arthritis. Mod. Rheumatol..

[25] Umegaki H (2016). Sarcopenia and frailty in older patients with diabetes mellitus. Geriatr. Gerontol. Int..

[26] Scott D, de Courten B, Ebeling PR (2017). Sarcopenia: A potential cause and consequence of type 2 diabetes in Australia’s ageing population. Med. J. Aust..

[27] Flahault A (2016). Low serum creatine kinase level predicts mortality in patients with a chronic kidney disease. PLoS ONE.

[28] Cooper R (2014). Body mass index from age 15 years onwards and muscle mass, strength, and quality in early old age: Findings from the MRC National Survey of Health and Development. J. Gerontol. A Biol. Sci. Med. Sci..

[29] Horio M (2015). Serum albumin, but not glycated albumin was a potent factor affecting the performance of GFR equation based on serum creatinine. Clin. Exp. Nephrol..

[30] Kochi M, Kohagura K, Shiohira Y, Iseki K, Ohya Y (2016). Inflammation as a risk of developing chronic kidney disease in rheumatoid arthritis. PLoS ONE.

[31] Sumida K (2018). Treatment of rheumatoid arthritis with biologic agents lowers the risk of incident chronic kidney disease. Kidney Int..

[32] Couderc M (2016). Prevalence of renal impairment in patients with rheumatoid arthritis: Results from a cross-sectional multicenter study. Arthritis Care Res. (Hoboken).

[33] Masson C (2011). Rheumatoid anemia. Jt. Bone Spine.

[34] Saisho K, Yoshikawa N, Sugata K, Hamada H, Tohma S (2016). Prevalence of chronic kidney disease and administration of RA-related drugs in patients with RA: The NinJa 2012 study in Japan. Mod. Rheumatol..

[35] Mori S, Yoshitama T, Hirakata N, Ueki Y (2017). Prevalence of and factors associated with renal dysfunction in rheumatoid arthritis patients: A cross-sectional study in community hospitals. Clin. Rheumatol..

