# Supplementary material for: Factors contributing to discrepant estimated glomerular filtration values measured by creatinine and cystatin C in patients with rheumatoid arthritis
Source: Sci Rep. 2021 May 10;11:9884. doi: 10.1038/s41598-021-89303-3 (PMC8110572; doi:10.1038/s41598-021-89303-3)
Supplement: Supplementary file 1 — Supplementary Tables. [file 41598_2021_89303_MOESM1_ESM.docx]

Factors contributing to discrepant estimated glomerular filtration values measured by creatinine and cystatin C in patients with rheumatoid arthritis

Akikatsu Nakashima^1^, Shigeto Horita^1^, Takahiro Matsunaga^1^, Ryo Inoue^2^, Takeshi Zoshima^3^, Ichiro Mizushima^3^, Satoshi Hara^3^, Kiyoaki Ito^3^, Hiroshi Fujii^3^, Hideki Nomura^4^, and Mitsuhiro Kawano^3*^

^1^Division of Nephrology and Rheumatology, Ishikawa Prefectural Central Hospital, Kanazawa, Japan

^2^Division of Internal Medicine and Rheumatology, Ishikawa-ken Saiseikai Kanazawa Hospital, Kanazawa, Japan

^3^Department of Rheumatology, Kanazawa University Hospital, Kanazawa, Japan

^4^Department of General Medicine, Kanazawa University Hospital, Kanazawa, Japan

Supplementary Table 1. Differences in parameters between Groups A, B_1_, and B_2_

| Parameter (units) | Group A (n=44) | Group B_1_ (n=156) | Group B_2_ (n=36) |
| --- | --- | --- | --- |
| Age (years) | 73.5±12.5^**^ | 65.7±12.5 | 53.2±13.7^**^ |
| Female | 34/10 (77.3%) | 123/33 (78.8%) | 31/5 (86.1%) |
| Disease Duration (years) | 17.9±14.2^*^ | 11.7±10.1 | 6.1±5.7^**^ |
| Body Mass Index (kg/m^2^) | 20.1±3.0^**^ | 22.6±3.8 | 21.8±2.6 |
| Hypertension , n (%) | 24/20 (54.5%)^**^ | 55/101 (35.3%) | 3/33 (8.3%)^**^ |
| Hyperlipidemia , n (%) | 9/35 (20.5%) | 31/125 (19.9%) | 2/34 (5.6%) |
| Diabetes Mellitus , n (%) | 15/29 (34.1%)^**^ | 19/137 (12.2%) | 4/32 (11.1%) |
| Proteinuria , n (%) | 3/40 (6.8%) | 13/141 (8.3%) | 1/35 ( 2.8%) |
| Hematuria , n (%) | 6/37 (14.0%) | 32/122 (20.8%) | 9/27 (25.0%) |
| DAS28-CRP | 2.23±1.14 | 2.04±0.87 | 1.52±0.75^**^ |
| DAS28-ESR | 3.16±1.32 | 2.72±0.99 | 1.97±1.01^**^ |
| RF (U/ml) | 160±236 | 122±294 | 181±636 |
| ACPA positive , n (%) | 22/11 (66.7%) | 90/32 (73.8%) | 23/10 (69.7%) |
| WBC ( /mm^3^) | 6,460±2,870 | 6,190±2,210 | 5,510±1,430 |
| Hb (g/dl) | 11.8±1.8^**^ | 12.8±1.4 | 13.0±1.3 |
| Plts. ( x 10^4^ /mm^3^ ) | 23.3±7.2 | 23.4±7.5 | 23.7±　6.1 |
| CRP (mg/dl) | 0.57±0.80 ^NS^ | 0.30±0.53 | 0.15  ±  0.36^**^ |
| ESR (mm/1hr.) | 43±31^**^ | 25±21 | 16±20^**^ |
| Albumin (g/dl) | 3.8±0.5^**^ | 4.1±.04 | 4.3±0.4 |
| BUN (mg/dl) | 18.4±6.2^*^ | 15.9±5.1 | 14.7±4.1 |
| Cr (mg/dl ) | 0.70±0.40 | 0.73±0.27 | 0.71±0.13 |
| CysC (mg/L) | 1.37±0.71^**^ | 1.00±0.31 | 0.76±0.14^**^ |
| eGFR-Cr (ml/min/1.73m^2^) | 77.5±25.4 | 71.1±19.3 | 72.9±15.2 |
| eGFR-CysC (ml/min/1.73m^2^) | 52.9±19.9^**^ | 73.1±20.9 | 99.7±22.0^**^ |
| CK (IU/L) | 63.1±36.0^**^ | 92.4±83.9 | 91.6±56.1 |
| Steinbrocker Stage 4 (n/%) | 21/23 (47.7%)^**^ | 30/126 (19.2%) | 1/35 (2.8%)^**^ |
| Steinbrocker Class 4 (n/%) | 10/34 (22.7%)^**^ | 2/153 (1.3%) | 0/35(0.0%)^NS^ |
| Methotrexate (n/%) | 16/28 (36.4%) | 84/72 (53.8%) | 20/16 (55.6%) |
| Prednisolone (n/%) | 29/15 (65.9%)^**^ | 70/86 (44.9%) | 9/27 (25.0%)^**^ |
| NSAIDs (n/%) p=0.066 | 6/38 (13.6%) | 49/107 (31.4%) | 10/26 (27.8%) |

^**^, p<0.01; ^*^, p<0.05

Bonferroni and Hochberg corrections were applied for post-hoc analysis of continuous and categorical data, respectively

Supplementary Table 2. Multinomial logistic regression for Groups A and B_2_ in reference to Group B_1_

|  | OR (95% CI) | |
| --- | --- | --- |
|  | Group A vs. B_1_ | Group B_2_ vs. B_1_ |
| Age (10 years) | 1.238 (0.819-1.871) | 0.554 (0.408-0.752) |
| Gender (female) | 1.997 (0.649-6.147) | 0.536 (0.148-1.944) |
| BMI (kg/m^2^) | 0.820 (0.712-0.945) | 0.901 (0.801-1.012) |
| Hb (g/dl) | 0.704 (0.529-0.937) | 1.092 (0.774-1.541) |
| CK (10 IU/L ) | 0.845 (0.748-0.956) | 1.039 (0.963-1.121) |
| NSAIDs | 0.261 (0.081-0.842) | 0.867 (0.346-2.170) |
| Diabetes Mellitus | 3.483 (1.284-9.447) | 1.486 (0.407-5.417) |
| Steinbrocker Stage 4 | 3.303 (1.314-8.299) | 6.253x10^-10^ |

Goodness of fit test, p=0.29
